# Impact of Biomaterials on Differentiation and Reprogramming Approaches for the Generation of Functional Cardiomyocytes

**DOI:** 10.3390/cells7090114

**Published:** 2018-08-21

**Authors:** Camilla Paoletti, Carla Divieto, Valeria Chiono

**Affiliations:** 1 Department of Mechanical and Aerospace Engineering, Politecnico di Torino, Corso Duca Degli Abruzzi 24, 10129 Turin, Italy; camilla.paoletti@polito.it; 2 Division of Metrology for Quality of Life, Istituto Nazionale di Ricerca Metrologica, Strada delle Cacce 91, 10135 Turin, Italy; c.divieto@inrim.it

**Keywords:** biomaterials, cardiomyocytes, differentiation, myocardial infarction, reprogramming

## Abstract

The irreversible loss of functional cardiomyocytes (CMs) after myocardial infarction (MI) represents one major barrier to heart regeneration and functional recovery. The combination of different cell sources and different biomaterials have been investigated to generate CMs by differentiation or reprogramming approaches although at low efficiency. This critical review article discusses the role of biomaterial platforms integrating biochemical instructive cues as a tool for the effective generation of functional CMs. The report firstly introduces MI and the main cardiac regenerative medicine strategies under investigation. Then, it describes the main stem cell populations and indirect and direct reprogramming approaches for cardiac regenerative medicine. A third section discusses the main techniques for the characterization of stem cell differentiation and fibroblast reprogramming into CMs. Another section describes the main biomaterials investigated for stem cell differentiation and fibroblast reprogramming into CMs. Finally, a critical analysis of the scientific literature is presented for an efficient generation of functional CMs. The authors underline the need for biomimetic, reproducible and scalable biomaterial platforms and their integration with external physical stimuli in controlled culture microenvironments for the generation of functional CMs.

## 1. Introduction

Myocardial infarction (MI) is caused by the obstruction of coronary arteries, resulting in the death of approximately 1 billion cardiomyocytes (CMs) in the left ventricle within a few hours, acute inflammation and degradation of the cardiac extracellular matrix (ECM), with the formation of a fibrotic scar. Fibrotic tissue is mechanically stiffer than healthy cardiac tissue, it is mainly populated by cardiac fibroblasts (CFs) and lacks beating CMs [1,2].

**Figure 1:**
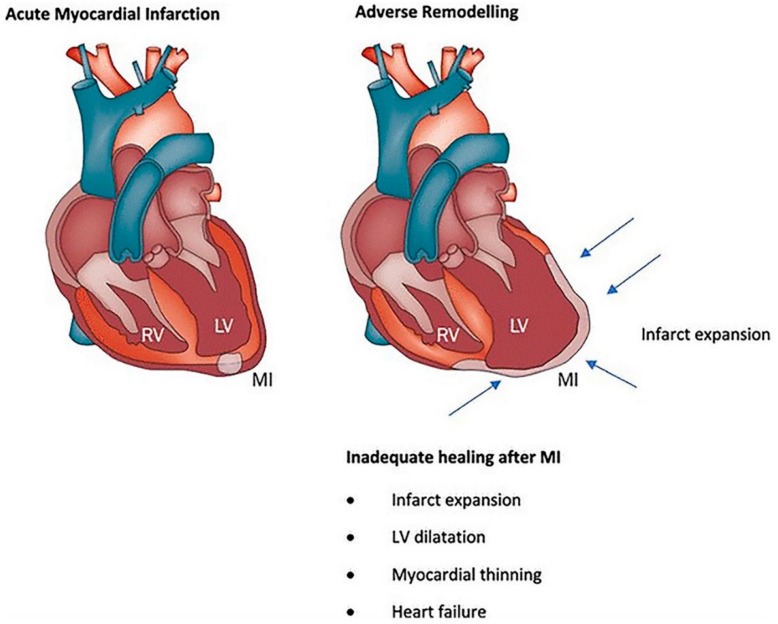
Left ventricular (LV) enlargement in the post-infarction phase due to thinning and dilation of the infarct zone (infarct expansion). Reproduced with permission from Curley et al. [3].

Despite significant advances in pharmaceutical and interventional therapies, the irreversible loss of functional CMs is still a critical issue. After ischemic heart disease, the dense collagenous scar replacing contractile CMs undergoes progressive negative remodelling, which impacts myocardial contractility and electrical conduction (Figure 1) [3].

This progressively leads to a severe deficiency of the ventricular pump function causing heart failure (HF) [4]. HF is the leading cause of mortality and morbidity in the industrialized world with more than 23 million cases worldwide and a survival rate of only 50% within 5 years from diagnosis [5,6]. For this reason, it has been defined as a global pandemic, accounting for 1–3% of all healthcare expenditures in North America, Western Europe and Latin America [7]. Nowadays, the only standard therapy for end-stage HF addressing the irreversible loss of CMs is heart transplantation, which however is limited by donor loss, risky surgical procedures and need for life-long immunosuppression.

In this scenario, there is a pressing need for new effective therapies for the functional regeneration of myocardial contractility, through the repopulation of the infarcted area with beating CMs. Cardiac patches, injectable hydrogels and cell therapy are under investigation as therapeutic approaches for myocardial regeneration [2,6,8,9]. However, cell therapy, consisting of the local infusion of cells at the infarct site, is constrained by poor cell retention and survival, which results in limited therapeutic benefit, mainly attributed to a ‘paracrine effect’ [10,11]. On the other hand, patches and hydrogels mechanically reinforce the fibrotic scar tissue and can also stimulate functional regeneration by the recruitment of endogenous cells or the delivery of exogenous cells [6]. The first strategy known as “endogenous cardiac regeneration” is limited by several constrains, including the low proliferative capacity of adult CMs, the limited regenerative potential of resident cardiac stem cells and the low number, poor homing ability and questionable in vivo differentiation into CMs of circulating bone-marrow derived stem cells [6]. On the other hand, in “exogenous cardiac regeneration” strategies, hydrogels and patches are applied as cell carriers and their biochemical, structural and mechanical properties may improve cell retention, viability, differentiation and integration with the host tissue compared to traditional cell therapies.

Furthermore, in the last decade, emerging studies have raised a new intriguing possibility for myocardial regeneration, represented by the direct reprogramming of fibroblasts into CMs. Considering the abundance of cardiac fibroblasts in the heart, the possibility for their direct reprogramming is expected to revolutionize therapies for myocardial regeneration by the direct conversion of dysfunctional fibrotic scar into contractile myocardial tissue [12,13,14,15,16]. Alternatively, the possibility to directly convert fibroblasts into CMs could also be exploited for in vitro generation of autologous CMs for the cellularization of implantable hydrogels and patches. In this review, we will describe the main available cell sources for CM generation, the experimental techniques to evaluate CM generation at the different levels (early, late, functional) of their maturation as well as the optimal biomaterial types inducing CM differentiation/reprogramming. Finally, we will critically discuss the key role of biomaterials in the functional maturation of CMs.

## 2. Cell Sources for the Generation of Cardiomyocytes 

Several cell sources can be employed for CM generation. Below, a brief description of pluripotent stem cells (embryonic stem cells, ESCs; induced pluripotent stem cells, iPSCs), adult stem cells (mesenchymal stem cells, MSCs and cardiac progenitor cells, CPCs) and direct/indirect reprogramming approaches is presented.

### 2.1. Embryonic Stem Cells (ESCs)

Embryonic stem cells are undifferentiated cells not yet committed to differentiate into any cell type; they reside within the inner cell mass of embryos at their blastocyst stage and are called blastomeres until the embryo is implanted into the uterus. Blastomeres can potentially differentiate into any adult cell type. However, after implantation into the uterus these cells initiate a differentiation process and reduce their pluripotency [17]. 

When blastomeres are extracted from the embryo and cultured under specific conditions in vitro, they can proliferate indefinitely by self-renewal to retain pluripotency [18]. On the other hand, under specific culture conditions, they can be differentiated in vitro to generate specific cell phenotypes. ESCs offer high potentialities for new drug testing, regenerative medicine, cell therapies, toxicity studies and disease systems modelling. ESCs can differentiate into CMs and are one of the most promising cell sources for cardiac regeneration. Evidences of heart regeneration from human ESCs have been demonstrated from studies in different animal models, such as pig, guinea pig and non-human primate models [17,19,20]. However, several ethical constrains hinder the use of ESCs for clinical research and applications. Firstly, the derivation of these cells from human embryos imposes an important ethical and legislative challenge. Secondly, ESCs have shown some potential risks due to their genetic instability and consequent tumorigenicity which, together with their potential immunogenicity, represent crucial limitations to their use [17]. These issues have been overcome by a new type of pluripotent stem cells, the induced pluripotent stem cells (iPSCs), described in the following paragraph.

### 2.2. Induced Pluripotent Stem Cells (iPSCs)

The iPSCs were firstly introduced by Takahashi and Yamanaka in 2006: they generated stem cells with similar properties to ESCs starting from adult differentiated cells [21]. In detail, they transduced skin fibroblasts with a combination of 4 reprogramming factors: Oct4 (Octamer binding transcription factor-4), Sox2 (Sex determining region Y)-box2, Klf4 (Kruppel Like Factor-4) and c-Myc. Through this approach, somatic cells were reprogrammed into iPSCs able to self-renew as well as to differentiate into several cell types, in analogy to ESCs. This discovery had a strong impact in the clinics and research, as for the first time it made available a population of autologous pluripotent stem cells and overcame the ethical issues of ESCs. 

Takahashi and Yamanaka’s work has been followed by the intense activity of a wide number of research groups, that proposed other reprogramming factors and/or methods to generate iPSCs with different efficiency levels. Singh et al. have recently reviewed the main methods to generate iPSCs [22]. iPSCs can be used in tissue regeneration, drug discovery and disease modelling. For tissue regeneration, iPSCs obtained from autologous somatic cells are differentiated in vitro and then transplanted at the injured site or degenerated tissue. A recent work published by Rojas et al. has shown that murine iPSC-derived CMs can be successfully engrafted in a murine model of myocardial infarction and such treatment improves murine heart function [23]. The iPSCs-based technology has also been applied to human cells, to generate human iPSCs for further differentiation into CMs [24]. Such approach makes autologous CMs available for cardiac regeneration and for the obtainment of patient- and/or mutation-specific models to study cardiac diseases and develop novel personalized therapeutic strategies. However, iPSC-derived CMs from human cells have shown limited functional maturation compared to adult human CMs, which may hinder their use in clinical applications. In general, as reported by Singh et al. the use of iPSCs is also mainly limited by safety concerns associated with the use of viral vectors for cell transfection, with possible risk for insertional mutagenesis after transfection [22]. Only once these limitations are overcome, it will be possible to exploit the enormous potentialities of iPSCs in the clinics.

### 2.3. Mesenchymal Stem Cells (MSCs)

MSCs are adult stem cells which reside in several mature tissues such as bone marrow, muscle, fat, synovial membrane, umbilical cord, peripheral blood, amniotic fluid, foetal liver and lung [25]. They are characterized by the capability of self-renewal through cell division and multipotency, showing the ability to differentiate under certain conditions into a variety of cell types, such as osteoblasts, adipocytes (fat cells), chondrocytes (cartilage cells), myocytes (muscle cells) for the regeneration of bone, fat, cartilage and muscle tissues, respectively. MSCs isolated from bone marrow (BMSCs) have been widely used in regenerative medicine applications due to their low immunogenicity and immunomodulatory effects, which minimize the immune rejection risk. These properties also make BMSCs ideal candidates for cardiac regeneration [26,27]. As reported by Miao et al. BMSCs offer some advantages for therapeutic treatment of cardiac disease, as they may support cardiac regeneration through paracrine effect, secreting several growth factors, such as cytokines like vascular endothelial growth factor (VEGF), fibroblast growth factor (FGF), hepatocyte growth factor (HGF), interleukin (IL)-6, platelet-derived growth factor (PDGF) and insulin-like growth factor (IGF) [27]. In addition, BMSCs may promote the recovery of cardiac function by inhibiting the inflammatory process. However, the use of BMSCs is associated with the potential risk to develop malignant spontaneous transformation, which has been observed mainly in in vitro cell cultures [28]. In addition, only few clinical trials exist to support the use of BMSCs for cardiovascular disease treatment: for instance, their clinical application would require the knowledge of the optimal cell dose supporting BMSCs viability after transplantation at the poorly vascularized infarct area. Other issues and concerns are related to poor cell engraftment, survival and differentiation of the injected adult stem cells.

### 2.4. Cardiac Progenitors Cells (CPCs)

CPCs reside within cell niches in atria, ventricles, epicardium and pericardium of the heart and are characterized by a strong heterogeneity [29]. According to the definition given by Mauretti et al. they are characterized by self-renewal ability and multipotency. Based on that, CPCs may differentiate into different cardiac cells such as cardiomyocytes, smooth muscle cells, endothelial cells and fibroblasts [29]. CPCs include different classes of stem cell subpopulations with partially overlapped phenotypic profiles: c-Kit (or CD117)-positive CPCs, discovered in mouse and then found also in human heart; cardiospheres and cardiosphere-derived cells (CDCs), clusters generating a great amount of proliferative cells; epicardium derived cells, the cardiac side population cells; stem cell antigen-1 CPCs; CPCs expressing Islet-1 (Isl-1); CPCs expressing platelet derived growth factor receptor-alpha (PDGFRα) [30].

A scientific debate has arisen on the role of CPCs on myocardial regeneration. Some researchers have reported that CPCs are not able to contribute to the formation of new cardiomyocytes [31,32]. On the contrary, other researchers have evidenced the possibility of heart regeneration after injury driven by different cell sources including myocytes, c-kit-positive bone marrow cells, c-kit-positive resident CPCs, cardiospheres, resident fibroblasts and tissue resident mesenchymal stem cells [33,34,35,36]. According to this view, CPC recruitment at the infarct area could in principle activate myocardial tissue regeneration. Alternatively, CPCs have also been transplanted into injured hearts resulting in improved cardiac function. In detail, several studies have demonstrated that CPCs are able to induce regeneration of injured heart in different animal models (mainly murine and rat models) [37]. The first clinical trial (the Cardiac Stem Cell Infusion in Patients with Ischemic Cardiomyopathy—SCIPIO) has also been performed and results have shown an improvement of the left ventricular systolic function and a reduction of infarct size [30]. More recently, van Berlo et al. have used genetic tracing experiments to follow CPC fate after injection and have demonstrated that minimal CMs are generated from c-Kit-positive CPCs in vivo [38]. In contrast, abundant cardiac ECs were derived from c-Kit-positive CPCs [38].

Hence, after many years of pre-clinical and clinical investigations, the limited knowledge on the molecular mechanisms regulating cardiac regeneration makes the debate on CPC role still open. Currently, based on the most recent findings, the main view is that adult stem cell therapy is responsible for a paracrine release of growth factors able to induce neovascularization or favourable changes in the cardiac scar, rather than the formation of new CMs.

### 2.5. Indirect and Direct Reprogramming

Yamanaka and Takahashi were the pioneers of cellular reprogramming [21]. The method they proposed was defined as indirect reprogramming, as an adult cell type may be reprogrammed into a new adult cell type by passing through an intermediate pluripotent (iPSCs) stem cell state, followed by differentiation into CMs.

On the other hand, in the direct reprogramming approach, the intermediate pluripotent stem cell stage is not present and one adult cell type can be converted into another adult cell type directly. Cardiac regeneration may be potentially achieved through direct reprogramming of non-myocyte cells (fibroblasts) into CMs by administering a set of transcriptional factors (TFs) and/or microRNAs (miRNAs) and/or small molecules [39,40,41]. Qian and Srivastava have recently reviewed in vitro and in vivo direct cardiac reprogramming approaches [39], while Chen et al. have provided an exhaustive review on direct reprogramming of mouse and human fibroblasts [40]. Different combinations of TFs and/or miRNAs and/or small molecules and different types of fibroblasts may result in different reprogramming efficiencies, evaluated by the expression of CM markers and electrophysiological and beating properties [40]. Factors mainly used to directly reprogram human cardiac fibroblasts into CMs are TFs, such as Gata4, Mef2c, Tbx5 and Hand2, able to induce rapid and efficient reprogramming of adult cardiac and dermal fibroblasts into cardiomyocyte-like cells [42,43]. Other epigenetic regulators, such as miRNAs and signalling proteins have shown their ability to regulate direct reprogramming [44]. Also, combinations of miRNAs can be used, as suggested by recent studies by Jayawardena et al. [45]. In detail, a set of four miRNAs (miR-1, miR-133, miR-208 and miR-499) has demonstrated the ability to directly reprogram fibroblasts into cardiomyocyte-like cells, showing CM properties and expression markers [45]. Moreover, the suppression of pro-fibrotic signals using small molecules has been reported to favour fibroblast reprogramming into CMs. Zhao et al. have shown that targeting the transforming growth factor-β (TGF-β) and Rho-associated kinase (ROCK) pathways in combination with exogenous expression of core cardiac TFs can improve fibroblast reprogramming into CMs with an efficiency up to 60% [43].

In addition to the possibility to directly convert fibroblasts into CMs, novel direct reprogramming approaches have been recently proposed to reprogram fibroblasts into induced CPCs, although less investigated [46]. One advantage of this approach is the proliferative behaviour of CPCs and their potential ability to differentiate into all the cell types populating the heart.

Direct cardiac reprogramming is one of the most recent approaches in the field of cell therapy applied to cardiovascular disease research and treatment. Even if it is at an early stage compared to other strategies, direct reprogramming presents several advantages, including the absence of ethical issues and the wide potential applications, from drug testing to disease modelling and regenerative medicine. However, further studies are needed to demonstrate the efficacy and safety of this strategy in animal models before translation into humans.

### 2.6. New Emerging Approaches for CM Generation

Studies performed on non-mammalian vertebrates, such as zebrafish, have demonstrated that this animal model possesses enhanced capacity for heart regeneration [47]. Zhang et al. have shown that cardiac regeneration in zebrafish, using a ventricle-specific ablation system, is driven by pre-existing cardiomyocytes, particularly through the trans-differentiation of atrial cardiomyocytes into ventricular ones [47]. Although this topic will not be discussed in detail in this review, these findings have raised the question if these cardiac regenerative strategies can also be induced in the human heart. In this context, Giacca et al. have been the pioneers in the study of methods to stimulate the proliferation of adult CMs [48,49]. They have found that CM behaviour is under the control of microRNA network: some endogenous microRNAs (e.g., miR-1) regulate CM expansion; others are able to induce the de-differentiation of CMs into a pluripotent phenotype (e.g., the miR-302/367 cluster); others hinder CM proliferation (e.g., the miR-15 family). Furthermore, some microRNAs have not a physiological role in CMs but when administered exogenously they affect CM proliferation (e.g., miR-199a-3p). Recently, Zacchigna et al. have also found that proliferating CMs are present in both maternal and foetal heart during pregnancy [50]. They have demonstrated that CM proliferation is regulated by a set of factors secreted by regulatory T cells: Cst7, Tnfsf11, Il33, Fgl2, Matn2 and Igf2 [50]. 

All these emerging studies are aimed at finding out a combination of therapeutics able to stimulate endogenous cardiac regeneration through the induction of adult CM proliferation.

## 3. Techniques for the Evaluation of the Formation of Functional Cardiomyocytes

In the last decades, the generation of CMs by cell differentiation and reprogramming approaches has spread out to meet the demand for specific therapies to treat MI. Although significant results have been obtained and CMs generated by such approaches display cardiomyocyte-related markers and functions, they are still far from full maturation. In this paragraph, the main characterization techniques allowing quantification of CM maturation are described. Peculiar adult CM characteristics can be grouped into five main categories: gene expression, cell morphology and structural organization, calcium handling, electrophysiological properties and oxidative metabolism [51].

One of the leading and easiest approaches to assess CM maturation is gene expression by RT-PCR analysis. In detail, the induction of cell commitment into cardiac lineage is suggested by the expression of Gata-4, Tbx5, Mef2c, Hand2 and Nkx2.5 TFs. During embryonic life, these TFs are essential for the correct differentiation and patterning of the heart and their loss of function causes human congenital heart disease [52]. Gata-4 is considered a pioneer cardiac TF, which opens chromatin structure in cardiac loci, acts jointly with the other TFs and enables subsequent activation of specific cardiac target genes [53]. The expression of these TFs can be observed in the first days of differentiation/reprogramming process in vitro and their different combinations have been frequently used to drive cardiac lineage commitment of stem cells and fibroblast reprogramming in vitro [43,54]. As CM maturation in vitro progresses, an increase in MHY7 expression, encoding for β-myosin heavy chain (β-MHC), relative to MYH6 expression, encoding for α-MHC, as well as the isoform switch of specific structural proteins, such as slow skeletal troponin I (ssTnI) to cardiac troponin I (cTnI) can be observed [55]. In addition, the activation of genes encoding for calcium handling, such as SERCA2 and CACNA1C and sodium/potassium channels, such as KCNJ12 indicates the in vitro differentiation toward a mature CM phenotype [56]. Despite most of these genes are present in CMs generated in vitro, their expression levels are lower than for adult CMs: key differences are mostly detected in cardiac ion channel and calcium handling genes, highlighting the immature phenotype of in vitro generated CMs [57].

Cell morphology and structural organization are also characterized to evaluate CM maturation. Adult CMs have a rod-shaped architecture and are aligned in culture, whereas CMs generated in culture have an irregular morphology and do not present cell alignment when cultured in vitro. Particularly, pluripotent stem cells-derived CMs have circular or triangular morphology and are significantly smaller compared to mature CMs [58]. Moreover, polyploidy is a characteristic of adult CMs: nearly 33% of adult CMs present more than one nucleus, whereas in vitro generated CMs are mostly mononucleated.

Sarcomere formation should also be evaluated [59]. Sarcomere is the fundamental unit for CM contraction and it is identified by the expression of contractile/myofilament proteins, such as α-sarcomeric actinin and β-MHC and regulatory proteins which modulate the activation of muscle contraction, such as tropomyosin and the cardiac troponin complex T (cTnT), C (cTnC) and I (cTnI) [60]. Both cellular (immunocytochemistry) and molecular (RT-PCR) -based analyses are used to evaluate the expression of these markers. For a deeper and complete study, sarcomere structure should be confirmed by electron microscopy. Adult CMs present a well-organized sarcomeric structure whose length reaches 2.2 µm (in relaxed adult human cardiac muscle cells), whereas iPSC-derived CMs have an immature sarcomeric organization of approximately 1.65 µm length [58].

Furthermore, calcium handling is important to determine CM maturation. Human adult CMs store calcium in the sarcoplasmic reticulum (SR). The commonly used markers to identify the presence of the SR are the regulatory proteins calsequestrin and phospholamban. Calsequestrin, involved in calcium binding and storage in the SR and phospholamban, which modulates SR Ca^2+^ sequestration, are expressed at abnormal levels or nearly absent in in vitro generated CMs [59]. Moreover, the formation of transverse tubules (T-tubules), which consist of membrane invagination along the Z-line regions of the cells, contributes to CM maturation. T-tubules allow synchronous triggering of SR calcium release and therefore, the propagation of cardiac action potential into the entire cytoplasm, thus initiating the process of excitation-contraction (EC) coupling [61].

Nowadays, the electrophysiology of in vitro generated CMs varies among different studies, depending on differentiation methods, cell types and culture times. Immature CMs have a higher resting membrane potential of about −60 mV compared to −90 mV displayed by mature CMs. Membrane depolarization in adult CMs is extremely fast (300 V/s in healthy hearts), whereas in vitro generated CMs show lower depolarization speed. The conduction velocity increases as the heart matures from 0.3 m/s to 1 m/s for neonatal and adult CMs, respectively. Zhu et al. have demonstrated that human ESC-CM conduction velocity is increased as in vitro cell maturation proceeds but still lower compared to embryonic CMs [62]. Additionally, the distribution of gap junctions is an important factor that regulates conduction velocity. During embryonic life, the gap junction protein Connexin 43 (Cx43) and the adhesion protein N-cadherin (N-cad) are distributed along the cell circumference, while in post-natal heart they tend to concentrate at the cell-cell junction, thus resulting in accelerated conduction velocity of CMs [63]. Wang et al. have shown that mouse iPSC-CMs cultured in vitro for 28 days express Cx43 in the cytoplasm but on the cellular membrane [64].

Finally, metabolic changes during CM maturation are important hallmarks that should be considered. During early stages of cardiac development, the major source of energy in embryonic CMs is glycolysis (nearly 80%). As CM maturation proceeds, cells switch their metabolic features to fatty acid-β oxidation, which generates a more energetic profile [55]. To satisfy the high energetic need, mitochondria occupy at least 30% of total adult CM volume [65]. Conversely, energy source in CMs obtained in vitro is based on glycolysis rather than on fatty acid–β oxidation, hence mitochondria are localized next to the nucleus or cell periphery instead of along myofibrils as in adult CMs. Zhou et al. have shown that iPSC-CMs generated from mouse cardiac fibroblasts displayed increased expression of genes related to glycolysis, whereas CMs induced by direct reprogramming of fibroblasts showed higher expression of genes involved in fatty acid-β oxidation [66].

In conclusion, the generation of mature and functional CMs in vitro that reflect the physiological characteristics of human adult CMs is difficult to be obtained and requires further progress. For this reason, different in vitro culture conditions that closely mimic the cardiac environment are under study to increase the yield of in vitro CM maturation.

## 4. Biomaterials for Guiding Cell Behaviour

Cell differentiation into a certain mature cell type is achieved in vivo by complex and multistep processes which are regulated by both intrinsic and extrinsic cell mechanisms [67,68]. Intrinsic factors are the genetic and molecular signatures expressed by the cells. Over the last decades, increasing evidences have suggested that also external stimuli from the surrounding microenvironment, including the extracellular matrix (ECM), contribute to the overall control of stem cell activity (extrinsic mechanisms) [69]. In detail, ECM stiffness, organization, biochemistry and embedded signalling molecules have been found to influence cell fate [70,71]. These signals are transduced intracellularly through specific interactions, mainly based on integrin receptors [72].

In the heart, ECM is a complex structure, which provides a crucial environment maintaining CM functions. Main cardiac ECM components include: glycosaminoglycans (GAGs) and proteoglycans as important structural molecules, different collagen proteins (collagen Type I, III and V) to provide elasticity and structural integrity to cardiac tissue and fibronectin, which interacts with integrins, GAGs and collagens to mediate cellular adhesion [73]. Several studies have reported in vitro stem cell differentiation into CMs on 2D tissue culture plates (TCPs). However, such approaches have generally failed in generating mature CMs, as in vitro generated CMs have generally shown characteristics closer to foetal than adult CMs [74]. Ideal culture conditions should not only support cell adhesion and growth but also provide an environment which mimics the native one where cells are able to express their in vivo phenotype features [75]. For this reason, traditional 2D cell culture methods have been overcome by new approaches. Currently, cardiac tissue engineering strategies are addressed to the development of 3D biomimetic substrates, which resemble the physiological environment for cell adhesion, proliferation and differentiation [76]. A variety of biomaterials have been used for the purpose, both from natural and synthetic origin [77]. Biomaterials can guide cell behaviour through their biochemical (composition) and physical (mechanical, electrical, structural) properties [78,79,80]. Particularly, in this work, the role of biochemical signalling exerted by different biomaterials on cell differentiation and reprogramming into CMs is highlighted.

Mostly used biomaterials for CM generation can be divided into three main categories:

(i) ECM-mimetic (mainly protein-based) biomaterials;
(ii) decellularized cardiac ECM;
(iii) “bioartificial” materials consisting of synthetic polymers functionalized with cardiac ECM proteins.

Biomaterials based on ECM components include: (i) cardiac ECM proteins, such as collagen and its derivative gelatin, laminin and fibronectin, isolated from animal or human tissues or derived from recombinant sources; (ii) natural polymer mixtures, such as Matrigel, a soluble basement membrane biomaterial extracted from Engelbreth-Holm-Swarm mouse tumours and Cardiogel, a cardiac ECM, deposited by in vitro culture of neonatal rat cardiac fibroblasts and consisting of collagen Type I and III, laminin and fibronectin [81,82]. However, obtaining a substrate that functionally resembles the intrinsic complexity of native cardiac ECM still represents a challenge [83].

Different studies have overcome this problem by using decellularized cardiac ECM [84]. Decellularized scaffolds offer an ideal environment, as they preserve the architecture of the whole organ as well as cell-binding domains, which are functional for cell adhesion, proliferation and migration [85].

Furthermore, several synthetic polymer-based substrates have been used for cardiac tissue regeneration in the last decades [86]. Synthetic polymer substrates can be easily fabricated and finely tuned to obtain scaffolds with specific mechanical properties for cell cultures, depending on the polymer type and scaffold architecture. Main synthetic polymers used in tissue engineering include: poly (ethylene glycol) (PEG), poly(lactic acid) (PLA), poly(glycolic acid) (PGA), poly(ε-caprolactone) (PCL) and their copolymers such as poly(lactide-co-glycolide) (PLGA) [8]. These polymers are biocompatible and biodegradable and approved by Food and Drug Administration (FDA) for medical use. New synthetic polymers may also be synthesized, including polyurethanes (PUs), block copolymers which physical and chemical properties may be tailored varying the composition [87]. For instance, an elastomeric PU scaffold has been recently developed by Chiono et al. as a substrate for CPC culture [88]. Synthetic biomaterials can be functionalized with ECM proteins or integrin-binding peptides as well as growth factors to improve cell attachment, proliferation and differentiation, obtaining “bioartificial” materials [89,90]. Indeed, Boffito et al. have shown that the surface functionalization of elastomeric PU scaffolds with the cardiac ECM protein laminin-1 enhances CPC proliferation as well as early differentiation into CMs, smooth muscle cells and endothelial cells and protects CPCs from apoptosis [91].

**Figure 2:**
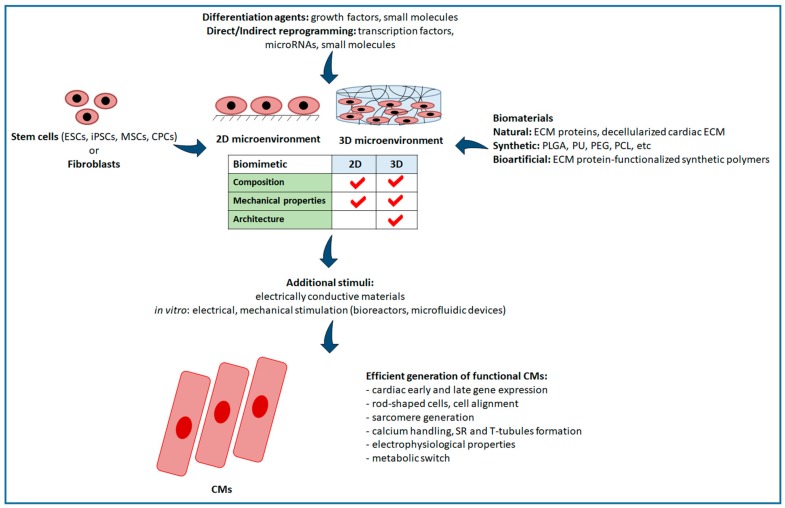
Schematic representation of CM generation. Functional and mature CMs can be obtained starting from different cell sources: through cardiac differentiation of stem cells (ESCs, iPSCs, MSCs and CPCs) or fibroblast direct and indirect reprogramming into CMs. These processes are mainly guided by differentiation agents (growth factors and small molecules) or reprogramming agents (TFs, microRNAs and small molecules). Cell differentiation and reprogramming can be performed in the presence of biomaterials, which are classified as natural (ECM proteins and decellularized cardiac ECM), synthetic (PLGA, PU, PEG, PCL, etc.) and “bioartificial” (ECM protein-functionalized synthetic polymers) using a 2D or 3D microenvironment. 3D cell culture offers a structure that mimics natural microenvironment. Additional stimuli for CM generation can be provided by electrically conductive materials or electrical and mechanical stimulation in bioreactors and microfluidic devices. Peculiar features of functional CMs assess the level of CM maturation.

In this section, we will review different biomaterial-based approaches improving differentiation of different stem cell types—ESCs, iPSC, MSCs and CPCs—as well as direct and indirect reprogramming of fibroblasts into functional mature CMs (Figure 2). Table 1 collects relevant examples of biomaterial substrates used for the differentiation of stem cells and the direct/indirect reprogramming of somatic cells to obtain cardiomyocytes, discussed in this critical review article.

### 4.1. Role of Substrate on Stem Cell Differentiation into CMs

#### 4.1.1. Biomaterials Supporting ESC Differentiation

**Figure 3:**
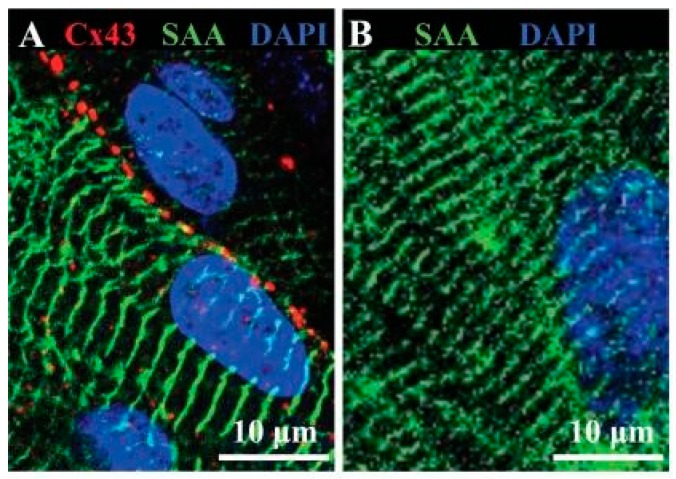
Representative immunostainings of hESC-CMs in 3D patches (**A**) and 2D monolayer (**B**). CMs in 3D patches present longer and more defined sarcomeres (α-sarcomeric actinin, green) compared to 2D culture after 2 weeks’ time. Moreover, 3D cardiac patches exhibit gap junctions Cx43 (red). Reproduced with permission from Zhang et al. Copyright 2018, Publisher Elsevier [92].

Zhang et al. have demonstrated that structural and functional maturation of human ESC-derived CMs can be enhanced using a 3D culture system [92]. They engineered a cellularized cardiac tissue patch by culturing human ESC-derived CMs, previously differentiated with an induction medium containing bone morphogenic protein 4 (BMP4), FGF and activin A, into a 3D hydrogel made of fibrin and Matrigel. After 2 weeks culture time, CMs in the 3D cardiac patch exhibited longer sarcomere structures and increased conduction velocity of action potential compared to cells cultured on 2D fibrin substrates. Moreover, the expression of contractile function-related genes, such as cTnT and α-MHC and excitation-contraction coupling genes, such as SERCA2 and CASQ2 was upregulated compared to 2D monolayers. In addition, Cx43 expression and the presence of gap junctions suggested functional electromechanical coupling between cells (Figure 3). The study demonstrated that a 3D biomimetic microenvironment can greatly enhance ESC differentiation into CMs.

Higuchi et al. have reported that mouse decellularized hearts supported mouse ESC (mESC) differentiation into CMs [93]. mESC-derived CMs, obtained with 5-azacytidine treatment, an established small drug that induces cardiac differentiation, showed superior expression of cTnI and α-MHC when cultured for two weeks on cardiac matrix compared to the case of mESCs cultured on liver decellularized ECM. Moreover, the expression of myosin light chain 3 (Mlc3), one of the myosin light isoforms involved in regulating cardiac muscle contraction, increased in mESCs cultured on cardiac ECM compared to liver ECM. Mouse decellularized heart has a different composition compared to liver decellularized matrix as it contains higher amounts of fibrillin-1, microfibrillar-associated protein 2 (Mfap2), microfibrillar-associated protein 5 (Mfap5) and lysyl oxidase homolog 1. In this study, a 3D ECM-based scaffold with biomimetic composition was found to better stimulate ESC differentiation into CMs.

Sa et al. have investigated the effect of fibronectin/laminin combinations on the differentiation of ESCs without using any induction molecule: they found out that fibronectin/laminin 70/30 caused the highest percentage of differentiation into CMs positive for Nkx2.5 and cTnI after 14 days culture time and proposed a mechanism based on integrin-mediated MEK/ERK signalling and direct cell-to-cell communication from distinct cells respectively expressing the laminin and fibronectin integrin receptors [95].

Prabhakaran et al. have mimicked cardiac ECM using electrospun PLGA/collagen Type I blend scaffolds, which promoted the formation of a spindle-like morphology in mESC-derived CMs compared to control culture conditions on TCPs [94]. Interestingly, cell proliferation increased on the blend substrate, with cells covering the entire scaffold surface, stimulated by the presence on the surface of the ECM protein collagen. Indeed, cells also showed cardiac structural maturation suggested by the expression of α-actinin and Cx43 after 5 days culture time compared to cells cultured on PLGA and control TCP substrates.

#### 4.1.2. Biomaterials Supporting iPSC Differentiation

Hirata et al. have coated the surface of polyacrylamide gels having different Young’s moduli (9, 20 and 180 kPa, coded as Es9, Es20 and Es180, respectively) as well as the surface of TCPs with ECM proteins, such as collagen Type I, fibronectin and gelatin to assess the effect of composition and surface elasticity on mouse iPSC differentiation into beating CMs [98]. While early cardiac differentiation genes such as Gata-4, Mef2c and Tbx5 were mostly upregulated on stiff TCPs immobilized with gelatin or fibronectin, the expression of cardiac contractile protein genes encoding for α-MHC, cTnT and cTnC was increased only in Es20 polyacrylamide gel immobilized with collagen 14 days after the initiation of differentiation. Interestingly, cardiac contractile genes were completely suppressed on iPSCs cultured on Es9-polyacrylamide gel, while the expression of stem cell markers, such as Nanog, was increased. Results suggested that both matrix elasticity and surface composition strongly affect cell differentiation.

Fong et al. have studied the influence of both 2D and 3D microenvironments consisting of foetal and adult bovine cardiac decellularized ECMs on human iPSC-derived CM maturation [97]. Initially, cardiac differentiation of iPSCs was induced regulating Wnt/Gsk3 pathway. 3D adult cardiac ECM, having high collagen content, and foetal cardiac ECM were found to enhance the expression of several genes related to cardiac maturation, including genes related to calcium handling (e.g., Junctin) and to inward rectifier potassium ion channels (KCNJ2/Kir2.1) after seven days culture time, compared to cells cultured on 2D cardiac ECM. Indeed, these genes were mostly upregulated in cells cultured on 3D adult ECM compared to cells cultured on foetal ECM. Moreover, 3D adult ECM induced a strong expression of cardiac maturation related genes compared to 2D culture conditions (Cav1.2 and Cx43) and 3D foetal ECM (myosin light chain 2, Mlc2), suggesting that both biochemical composition and architecture influence human iPSC-derived CMs maturation.

**Figure 4:**
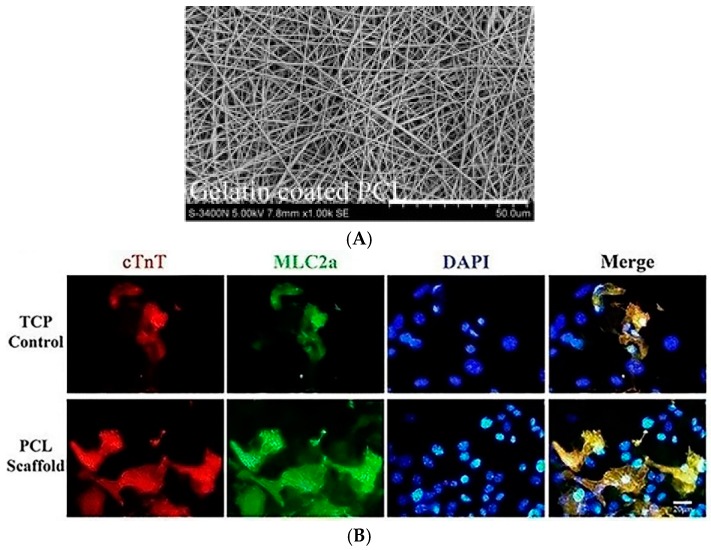
Representative image of poly-(ε-caprolactone) (PCL) nanofibrous scaffold coated with gelatin (upper panel, (**A**)) used to promote CM differentiation of miPSCs. Gelatin-coated PCL scaffolds support miPSC differentiation into CMs, increasing the expression of cTnT and Mlc2a after 15 days culture time (lower panels, (**B**)). Reproduced with permission from Chen et al. [96].

Chen et al. have evidenced the effects of 3D gelatin-coated PCL nanofibrous scaffolds on the in vitro differentiation of iPSCs derived from mouse adult fibroblasts into CMs (Figure 4) [96].

After 15 days culture time, cells seeded on gelatin-coated PCL electrospun scaffolds showed increased expression of the early mesoderm marker MESP1 (basic helix-loop-helix transcription factor mesoderm posterior 1), early cardiac markers Gata-4 and Nkx2.5 and the late cardiac-specific gene for cTnT compared to cells cultured on TCPs. Moreover, the activation of Wnt/β-catenin signalling in cells cultured on the 3D PCL nanofibrous scaffolds influenced iPSCs differentiation into CMs during the early stage of the differentiation process.

#### 4.1.3. Biomaterials Supporting MSC Differentiation

Santhakumar et al. have used Cardiogel for the differentiation of mouse MSCs into cardiac lineage [101]. Cardiogel has been previously found to improve CM growth and maturation [111]. Mouse heart-derived Cardiogel not only supported mouse bone marrow-derived stem cells (BMSC) proliferation but also enhanced their cardiac commitment without the addition of external growth factors or chemical inducers. Approximately 15–20% of mouse BMSCs cultured for 4-weeks on Cardiogel developed three-dimensional myotubule-like multinucleated structure. Cardiac cell commitment was further confirmed by the expression of Gata-4, α-sarcomeric actinin and Cx43.

Recently, MSCs have been cultured on 3D collagen Type I scaffolds—already used in clinical trials for cardiac repair—as an in vitro model of cardiac tissue [104]. MSCs on collagen patches showed increased expression of cardiomyocyte-specific proteins (although, cells did not reach a complete functional maturation), secreted higher levels of cardiotrophic factors and developed a less myofibrogenic phenotype. Additionally, when stimulated with either TLR3 or TLR4 agonists, MSC expression of pro-inflammatory cytokines was decreased in the presence of the collagen patch. However, in this study the main effect of collagen sponges on MSC behaviour was attributed to their tri-dimensionality and biomimetic mechanical properties rather than to their biochemical properties. Indeed, in previous studies using TCPs pre-coated with collagen Type I or laminin and non-coated TCPs, human MSCs have shown a similar expression of lineage-specific (cardiogenic, osteogenic and adipogenic) genes, while the cardiogenic gene expression has been enhanced by MSC culture in 3D collagen Type I substrates [103]. Similarly, another study has demonstrated that collagen Type I substrates in the form of hydrogel, sponge, or membrane have a different effect on the immunosuppressive and paracrine behaviour of MSCs [112]. These studies have suggested that collagen Type I does not provide specific biochemical cues to promote MSC differentiation into CMs.

Tan et al. have investigated the effect of collagen Type I, III and V matrices and integrin activation on the regulation of human BMSC commitment to CMs [99]. BMSC-derived CMs cultured in a myogenic differentiation medium on uncoated TCPs showed increased expression of collagen-binding α2β1 and α5β3 integrin receptors suggesting that cell interaction with ECM is crucial for cell differentiation. Indeed, CMs showed enhanced cell adhesion and proliferation after 1 day when cultured on collagen Type V-coated TCPs compared to either uncoated TCPs or other collagen Types (I and III). Collagen Type V matrices promoted the expression of both early cardiac genes, such as Gata-4 and Nkx2.5 and downstream genes, such as Ryanodine receptor 2(RyR2) involved in calcium release, α-skeletal actinin, cTnT and cTnC. Moreover, cardiac cells generated on collagen Type V matrices prevented chamber dilatation and improved contractile function when injected in vivo into the injured myocardium of mouse subjected to MI.

Later, Guan et al. have hypothesized that mimicking 3D cell alignment of myocardium could enhance cell differentiation [100]. They induced MSC differentiation into cardiac lineage by a tissue construct engineered by simultaneous electrospinning of poly(ester carbonate urethane)urea (PECUU) fibres and electrospray of human MSCs. Electrospinning allowed the obtainment of elastomeric nanofibers mimicking the size and aligned organization of collagen fibres in the myocardium. Cells integrated in the nanofibrous scaffold showed enhanced cardiac differentiation with a significant increase in Mef2c and Nkx2.5 gene expression and a strong upregulation of Gata-4 expression (more than 700 times) compared to monolayer culture, suggesting that cardiac-like structural microenvironment can induce cardiac differentiation of MSCs. However, a more pronounced gene expression was achieved by subjecting scaffolds to mechanical stretching during in vitro culture.

The field of tissue engineering have further investigated the role of other kinds of materials in regulating cardiomyogenic differentiation of MSCs, such as electrically-conductive graphene-based substrates. Park et al. have found that cardiomyogenesis-related gene expression is increased in human MSCs cultured on graphene substrates compared to cells seeded on coverslips [102]. Particularly, graphene increased the expression of the early cardiac transcription factor Gata-4 and other cardiac-related structural and functional proteins such as β-MHC, cardiac actinin, cTnT, myosin light chain 2A (Mlc2a) and 2v (Mlc2v) and Cx43 after 2 weeks culture time. However, CMs generated on graphene substrates did not show electrophysiological functions, suggesting that additional signals are required for complete CM maturation.

#### 4.1.4. Biomaterials Supporting CPC Differentiation

French et al. have used a decellularized porcine derived-cardiac ECM to examine its ability to support CPC in vitro differentiation compared to collagen matrix [105]. Culture of CPCs isolated from rat on cardiac ECM showed increased gene expression of early CM markers such as Nkx2.5, cTnT and α-MHC and a decreased expression of fibroblast markers such as fibroblast-specific marker 1 (FSP-1) after two days culture time. In addition, cells seeded on cardiac ECM showed higher Gata-4 protein level after seven days culture time compared to cells on a collagen matrix. CPCs also showed increased proliferation and adhesion and a reduction in apoptosis when cultured on cardiac ECM compared to those cultured on collagen matrix.

Gaetani et al. have encapsulated human CPCs, isolated from both foetal and adult patients, in 3D hydrogels made of collagen Type I or lyophilized decellularized porcine ventricular ECM [107]. Despite both hydrogel types supported foetal and adult cell viability, only myocardial ECM hydrogel induced significant increase of cardiac gene expression in both foetal CPCs, enhancing expression of Gata-4, Mlc2v and the vascular marker VEGFR2 and in adult CPCs, with a significant increase in Nkx2.5, Mef2c, CD31 and VEGFR2, after 4 days culture time. CPCs encapsulated in myocardial ECM hydrogel also showed increased cell proliferation compared to collagen hydrogel. Moreover, cell-encapsulated hydrogel based on myocardial matrix could be formed in vivo after injection into the ventricular wall of healthy rats, representing a promising system for further in vivo studies.

Castaldo et al. have described a method for in vitro production of a human biomatrix, obtained by adult human cardiac fibroblasts isolated from both healthy and pathological hearts, as a substrate for culturing human CPCs in vitro [106]. Biomatrix from pathological patients showed increased content in fibronectin and collagen, which deposition is responsible for scar formation. Biomatrix from healthy patients stimulated CPCs migration, while both normal and pathological heart-derived biomatrix protected CPCs from apoptotic processes.

Chiono et al. have recently prepared a three-dimensional biocompatible scaffold by melt-extrusion additive manufacturing based on a synthesized poly(ester urethane) (PU) with elastomeric-like behaviour for myocardial tissue applications [88]. The PU scaffold supported in vitro human CPC adhesion and viability, however it could not stimulate CPC proliferation. For this reason, the PU scaffold was then surface grafted with a biomatrix protein, laminin-1 and a general adhesion protein, gelatin [91]. Laminin-1 functionalization could increase CPC proliferation and protect the cells from apoptosis after 7 and 14 days culture time. Additionally, RT-PCR analysis demonstrated the enhanced expression of cardiomyocyte (Mef2c and α-sarcomeric actinin), smooth muscle cell (Gata-6 and SMA) and endothelial cell (ETS1 and FVIII) markers on laminin-1 grafted PU scaffolds especially after 7 days culture time. This result suggested that the scaffold surface composition alone was able to stimulate early CPC differentiation compared to control substrates (PU and gelatin coated PU scaffolds).

### 4.2. Role of Substrate on Fibroblast Direct and Indirect Reprogramming into CMs

Direct as well as indirect reprogramming of fibroblasts into CMs may benefit from the use of ECM-mimetic substrates to control cell conversion into CMs. One demonstration of the importance of cardiac microenvironment in cardiac fibroblast direct reprogramming into mature CMs was provided by in vivo heart delivery of the TFs Gata-4, Mef2c and Tbx5 (GMT), which improved the direct reprogramming efficiency of cardiac fibroblasts into CMs, 4 weeks after MI, compared to 2D in vitro culture [113].

Kong et al. have also assessed that indirect reprogramming of MEFs into CMs is influenced by ECM [108]. MEFs, transduced with polycistronic Oct4-Sox2-Klf4-cMyc lentiviral vector (OSKM) to obtain progenitors cells and subsequently supplemented with BMP4 to induce cardiac differentiation were cultured on different natural ECM protein-based hydrogels, such as Matrigel, collagen Type I and fibrin. Fibrin gels enhanced nearly 4 times the dedifferentiation phase (indicated as the total number of colonies per cm^2^) after 7 days culture time and the subsequent differentiation step after 15 days culture time (indicated as percentage of contractile colonies), compared to Matrigel and collagen Type I hydrogels. Furthermore, blend hydrogels based on collagen Type I and fibrin induced similar reprogramming outcomes to pure fibrin hydrogels, while the percentage of contractile colonies out of the total number of cell colonies was directly proportional to collagen Type I content.

The role of biomaterials in supporting CMs generation has also been investigated in direct reprogramming approaches. Smith et al. have used PEG hydrogels as a substrate for cell reprogramming because of its ability to be functionalized with precise concentrations of adhesion molecules or short peptides avoiding unwanted protein absorption [109]. Cells were cultured in a medium containing Jak-1 inhibitor in the first step of direct reprogramming and BMP4 for the progress of direct reprogramming. PEG hydrogels functionalized with high laminin and arginine-glycine-aspartic acid (RGD) peptide concentrations supported Yamanaka factor-mediated MEF reprogramming, showing higher number of beating cells after 18 days culture time, compared to TCPs or PEG functionalized with one bioactive molecule (laminin or RGD), or with laminin and RGD at lower concentrations. Moreover, RT-PCR showed that laminin-functionalized PEG hydrogels induced higher upregulation of α-sarcomeric actinin and cTnT expression and lower expression of pluripotency marker Nanog compared to MEFs cultured on TPCs coated with laminin.

**Figure 5:**
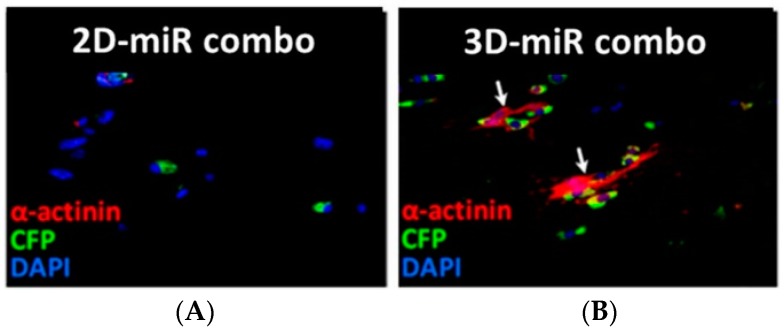
Representative immunostainings of neonatal cardiac mouse fibroblasts transfected with miR combo on 2D (**A**) and 3D (**B**) environment. 3D fibrin-based hydrogels (right panel) supported fibroblast reprogramming into CMs increasing α-sarcomeric actinin expression compared to 2D culture (left panel) after 14 days culture time. Reproduced from Li et al. [110].

Recently, the crucial role of biomimetic biomaterials in regulating cell fate has been investigated in the direct reprogramming of fibroblasts into cardiomyocyte-like cells using miRNAs [110]. Li et al. have improved miR combo (miR-1, miR-133, miR-208 and miR-499) mediated direct reprogramming process of neonatal murine cardiac fibroblasts using a fibrin-based 3D hydrogel containing Matrigel [110]. Fibroblasts transfected with miR combo and encapsulated in 3D hydrogels showed enhanced mRNA levels of several cardiac genes such as α-MHC, cTnI, α-sarcomeric actinin and Kcnj2 compared to fibroblasts cultured on traditional 2D culture dishes (Figure 5).

Similarly, immunostaining analysis demonstrated that the number of positive cells for cTnT was increased in 3D versus 2D culture condition. Moreover, the expression of early cardiac TFs was upregulated in both neonatal cardiac fibroblasts, with increased Mef2c expression and in neonatal TTFs, showing increased Mef2c, Tbx5 and Hand2 expression, in 3D compared to 2D cell culture environment. Furthermore, reprogramming process in 3D hydrogels was shown to be a mechanism dependent on metalloproteinases (MMPs), which are commonly upregulated in infarcted hearts.

## 5. Critical Analysis of the State of the Art and Future Perspectives

Stem cell differentiation is generally performed by combinations of soluble factors such as growth factors and low molecular weight drugs, while direct and indirect reprogramming of somatic cells is mainly performed by cell transfection with TFs and/or microRNAs via viral or non-viral vectors to modify gene expression [114,115]. However, cell-ECM interplay, mediated by the interactions of cell integrin receptors to peptide motifs in ECM proteins has a well-known role in directing tissue-specific cell functions such as cell adhesion, proliferation, survival, differentiation and cytoskeleton organization. Traction forces are exerted through integrin receptor-ligand interactions affecting cell shape and intracellular signalling cascade regulating gene expression. Hence, the interplay between cells and their substrate may strongly contribute to stem cell fate and cell reprogramming. Indeed, the induction of differentiation or reprogramming of cells cultured on a suitable biomaterial substrate may enhance the efficiency and speed of cell conversion and increase the level of cell functional maturation. The substrate may affect cell differentiation and reprogramming through its biomimetic biochemical properties and architecture, resulting in tissue-specific mechanical properties. In general, 3D compared to 2D substrates are preferred as they more closely mimic the native ECM microenvironment. Based on that, optimal biomaterial substrates should mimic the chemical and mechanical properties of the ECM naturally hosting all the cells involved in the differentiation/reprogramming route. Hence, an ideal substrate for differentiation and reprogramming should combine multiple chemical cues (i) allowing cell viability, attachment and proliferation of starting cells (stem cells or fibroblasts); (ii) favouring cell differentiation or reprogramming and (iii) enhancing the attachment and functional maturation of cardiomyocytes. In more detail, in the case of pluripotent or multipotent stem cells, biomaterials should support adhesion, proliferation and differentiation of cells as they gradually lose their potency, finally improving the maturation of differentiated CMs. In the case of indirect reprogramming, the substrate should favour fibroblast as well as pluripotent stem cell attachment and proliferation and provide the biochemical and mechanical cues favouring the differentiation and maturation process. Finally, in the case of direct reprogramming of fibroblasts into CMs, biomaterials should allow fibroblast attachment and proliferation as well as CM attachment and functional maturation. Based on the literature analysed in this critical review article, Table 2 collects biomaterial substrates showing favourable biochemical cues for enhanced stem cell differentiation or fibroblast direct/indirect reprogramming into CMs.

Table 2 shows that similar substrates have been used to induce ESC and iPSC differentiation into CMs, such as decellularized cardiac ECM, fibrin-based hydrogel, 3D synthetic scaffolds/hydrogels coated with collagen Type I or gelatin [92,93,94,95,96,97,98]. A recent work by Hirata et al. has shown that biomimetic stiffness of 3D substrates compared to myocardial tissue (20 kPa) is required to support the expression of cardiac contractile protein genes during pluripotent stem cell differentiation [98]. Hence, biomimetic chemical composition and 3D architecture should result in biomimetic mechanical properties for enhanced support to the differentiation and maturation process.

Similarly, for adult stem cells, a 3D microenvironment providing biomimetic chemical and mechanical cues may enhance differentiation potential. However, due to the different features (potency and integrin expression) of pluripotent and multipotent stem cells, specific chemical cues should be employed. Among multipotent stem cells, MSCs have been commonly differentiated into osteoblasts, chondrocytes, myocytes and adipocytes [25]. However, their plasticity has also been exploited to generate other cell types, such as CMs, under the stimulation of a proper culture microenvironment. For instance, MSCs have been differentiated into CMs when cultured on Cardiogel or collagen Type V substrates [99,101]. On the other hand, Collagen Type I and laminin have not shown specific biochemical cues able to induce MSC differentiation into CMs [103,104]. However, 3D substrates based on collagen Type I with similar mechanical properties to the native myocardium have enhanced MSC differentiation into CMs [104]. Considering MSC plasticity and their possibility to differentiate into different cell types, biochemical cues should be properly combined with other cues, such as substrate biomimetic stiffness, electrically conductive materials (e.g., graphene-based composites) and dynamic cell culture conditions (e.g., mechanical stretching) to promote MSC differentiation into CMs [100,102].

Compared to MSCs, CPCs are naturally committed to regenerate myocardial tissue. CPC differentiation into CMs has been favoured by culturing the cells on decellularized heart ECM, biomatrix-based substrates, as well as on scaffolds functionalized with laminin-1 [91,105,106]. Laminin-1 is the laminin form predominating in the heart tissue during early embryogenesis and further organogenesis and it is re-expressed in heart regeneration following tissue damage [116,117]. Studies on the interaction of CPCs with cardiac ECM constituent proteins have shown that laminin-1 protects CPCs from apoptosis and stimulates their proliferation [117]. Recent studies have also demonstrated that laminin-1 enhances CPC differentiation [91]. Hence, it represents an ideal biomimetic protein for stimulating CPC viability, proliferation, as well as differentiation into CMs as an alternative to complex cardiac ECM-based substrates.

Finally, fibroblast direct reprogramming is one of the most recent strategies investigated for CM generation. Despite the early promising results, the direct reprogramming of human adult fibroblasts into mature CMs still represents a challenge [40]. Only few studies have investigated the role of 3D biomaterial substrates, such as fibrin/Matrigel hydrogels and PEG-based hydrogels containing laminin and RGD, in direct reprogramming of fibroblasts into CMs [109,110]. Such studies have evidenced that the reprogramming efficiency and CM maturation may be significantly improved in 3D biomimetic substrates. On the other hand, several studies have demonstrated that traditional 2D in vitro tests have underestimated the efficiency of direct reprogramming compared to in vivo trials in mouse model [14]. This suggests that 2D TCPs fail in recapitulating the complexity of the native cardiac tissue and indirectly demonstrates that 3D biomimetic substrates may enhance direct reprogramming. Based on that, 3D in vitro models of human cardiac tissue are demanded to predict in vivo direct reprogramming efficiency. Additionally, 3D biomimetic substrates may represent a valid tool to enhance both in vitro and in vivo direct reprogramming of fibroblasts into CMs and to increase the functional maturation level of CMs, greatly improving the clinical potentialities of the direct reprogramming approach. 

Among the different strategies for myocardial regeneration and treatment, the use of iPSCs as cellular source is promising as it would allow patient-specific therapy as well as in vitro modelling of cardiac tissue. Similarly, direct reprogramming of human fibroblasts into CMs deserves future investigation as a new intriguing possibility for MI treatment by the *in situ* conversion of fibrotic scar (populated by cardiac fibroblasts) into contractile tissue. Additionally, direct reprogramming approach may also be exploited for CM generation in vitro for cardiac tissue modelling as well as cell therapy. 

**Figure 6:**
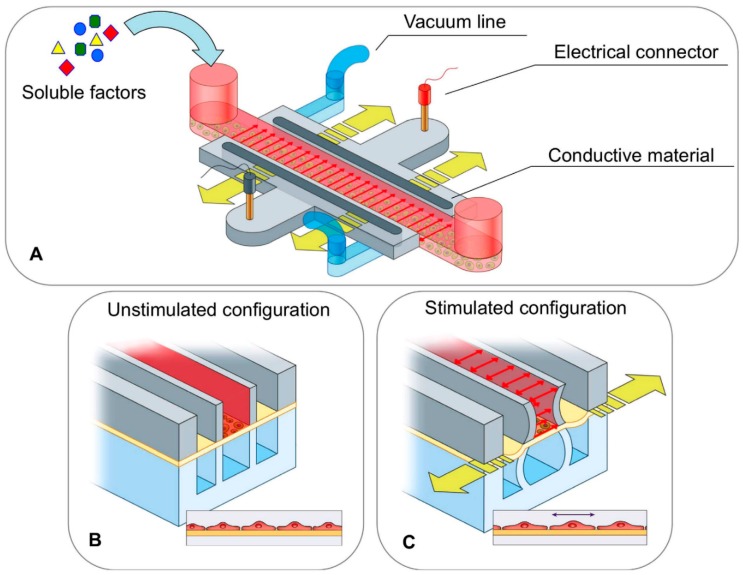
Microfluidic platform developed to study the effect of biochemical, mechanical and electrical stimulations for stem cell differentiation: (**A**) Schematic view; (**B**) cross-section view in unstimulated configuration; (**C**) cross-section view in stimulated configuration. The central channel (in red) is the media channel providing nutrients and soluble factors to cells. The pneumatic channels (in light blue) perform mechanical stimulation by stretching the poly(dimethylsiloxane) (PDMS) membrane (yellow arrows) where the cells are cultured. The electrically conductive layer (in light grey) is based on two regions composed of PDMS and carbon nanotubes (CNT) mixture, connected to the stimulator by two external gold-coated connectors (in red and black). Reproduced from Pavesi et al. [5].

Biochemical cues represent important stimuli to promote the formation of fully mature human adult CMs with high efficiency. One effective method to discriminate the influence of biochemical composition on cell differentiation and reprogramming is to perform in vitro differentiation and reprogramming experiments on 2D TCPs coated with different biomolecules. Indeed, such experiments allow the selection of biomimetic molecules for the subsequent production or functionalization of 3D scaffolds aimed at inducing stem cell differentiation or fibroblast reprogramming into CMs. On the other hand, 3D scaffolds combine biochemical, structural and mechanical cues which in synergy affect cell behaviour. Additionally, bioreactors and microfluidic devices (Figure 6) may be used for the in vitro culture of cells on scaffolds or hydrogels under mechanical and/or electrical stimulation, in dynamic flow conditions simulating the native microenvironment [118].

Such physical stimulation may further enhance differentiation and reprogramming into CMs. Particularly, biomimetic high-throughput microfluidic devices have been recently proposed to study stem cell differentiation in dynamic conditions in the presence of biochemical, electrical and mechanical cues [5]. Such devices have the inherent advantage to make use of a moderate number of cells and to provide a biomimetic culture microenvironment for the selection of hydrogels with proper chemical and mechanical properties for stem cell differentiation into CMs. Additionally, microfluidic systems are compatible with the common techniques used for evaluating cell differentiation or reprogramming such as immunocytochemistry and RT-PCR analysis. In the next future, microfluidic systems could be extended to the study of direct reprogramming of fibroblasts into CMs, representing a valid tool for the selection of proper biomaterial substrates and physical stimuli (e.g., mechanical stretching and electrical stimulation) for the efficient fibroblast conversion into mature CMs.

Interestingly, the wide knowledge available from experiments on stem cells differentiation into CMs could be effectively extended to the new of field direct reprogramming, speeding up the maturation of such promising technique in the perspective of future clinical applications. 

As a conclusion, generation of mature human adult CMs from stem cell differentiation or fibroblast reprogramming still represents a challenge and further studies are needed to understand the processes underlying the low efficiency of cell conversion, as well as the incomplete maturation of CMs. A multidisciplinary approach involving the combination of multiple cues in the cell microenvironment may represent a valid tool to find out the optimal conditions for stem cell differentiation and fibroblast reprogramming into CMs.

## 6. Conclusions

The field of cardiac tissue engineering is rapidly evolving to find out regenerative therapies to treat myocardial fibrosis and dysfunction after MI. Among the possible routes, indirect and direct fibroblast reprogramming strategies into CMs represent interesting approaches and deserve future investigation in the perspective of possible clinical applications. Biochemical composition of the culture substrate represents one valid tool to overcome the low conversion efficiency into CMs and the low maturation stage of CMs generated by differentiation or direct reprogramming approaches. Indeed, knowledge arising from previous studies on the differentiation of ESCs and adult stem cells can be exploited to engineer biomimetic 3D substrates for efficient indirect and direct fibroblast reprogramming into mature CMs. Human or animal-derived ECM materials, such as decellularized cardiac ECM, Cardiogel, Matrigel and biomatrix have been frequently used for stem cell differentiation [2]. Although such complex materials have been found to enhance stem cell differentiation into CMs, their allogenic or xenogenic nature makes them potentially immunogenic. Additionally, natural materials, including collagen and fibrin, suffer from limitations such as batch-to-batch variation, low mechanical resistance and fast degradation rate. On the other hand, “bioartificial” substrates in the form of synthetic scaffolds or hydrogels functionalized with adhesive peptides or biomimetic proteins may represent a valid alternative, thanks to their reproducibility, scalability and biomimicry. Furthermore, synthetic polymer chemistry, hydrogel crosslinking degree and scaffold architecture may be easily tailored to obtain 3D substrates with biomimetic mechanical stiffness and geometry, which together with the surface chemical composition may act in synergy to promote efficient indirect and direct reprogramming into functional CMs. Finally, a full micro-environmental control of the differentiation and reprogramming process in terms of biochemical, bio-mechanical and electrical cues is demanded to further stimulate maturation and structural organization of the cardiac micro-tissue deriving from differentiation or reprogramming approaches.

## Figures and Tables

**Table 1:** Influence of different biomaterial substrates on stem cell differentiation and fibroblast (direct and indirect) reprogramming into cardiomyocytes.

Cell Types	Substrate Type	Main Results	References
hESC	3D fibrin/Matrigel hydrogel cardiac tissue patch vs. 2D fibrin/Matrigel culture	Longer sarcomere length, increased action potential conduction velocity, expression of cTnT, α-MHC, SERCA2, CASQ2 and Cx43	Zhang et al., 2013 [92]
mESCs	Mouse decellularized heart ECM vs. liver ECM	Increased expression of cTnI, α-MHC and MCL3	Higuchi et al., 2013 [93]
mESC	Collagen Type I blended PLGA electrospun scaffold vs. PLGA and TCP	Acquisition of a spindle-like cardiomyocyte morphology, expression of α actinin and Cx43	Prabhakaran et al., 2014 [94]
hESC	Fibronectin (70%) and laminin (30%) combination substrate	Generation of 60% of cTnI and Nkx2.5 positive cells	Sa et al., 2014 [95]
miPSC	3D gelatin-coated electrospun PCL scaffold vs. TCP	Increased expression of MESP1, Gata-4, Nkx2.5 and cTnT	Chen et al., 2015 [96]
hiPSC	2D vs. 3D bovine decellularized cardiac foetal and adult ECM	3D adult ECM enhanced genes related to calcium handling (JNC), inward rectifier potassium ion channels (KCNJ2/Kir2.1), CaV1.2, Cx43 and Myl2	Fong et al., 2016 [97]
miPSCs	Polyacrylamide gels with different elastic moduli and functionalized with collagen Type I, fibronectin and gelatin vs. TCP	Expression of Gata-4, Mef2c and Tbx5 increased on gelatin/fibronectin-TCP; α-MHC, cTnT and cTnI increased on Es20 collagen gel.	Hirata et al., 2018 [98]
hBMSCs	Collagen Type V matrix	Expression of both early cardiac genes, such as Gata-4 and Nkx2.5 and their downstream genes, such as α skeletal actinin, cTnT and cTnC.	Tan et al., 2010 [99]
hMSCs	3D tissue PECUU fibrous construct vs. TCP	Cardiac differentiation with increased expression of Mef2c, Nkx2.5 and Gata-4	Guan et al., 2011 [100]
mMSCs	Cardiogel (fibroblast-derived ECM enriched in collagen Type I and III, laminin and fibronectin)	Approximately 15–20% of mouse BMSCs cultured for 4-weeks on Cardiogel developed three-dimensional myotubule-like multinucleated structure. Cardiac cell commitment was further confirmed by the expression of Gata-4, α-sarcomeric actinin and Cx43.	Santhakumar et al., 2014 [101]
hMSCs	Graphene substrate	Enhanced Gata-4, β-MHC, cTnT, Mlc2a, Mlc2v and Cx43 expression.	Park et al., 2014 [102]
hMSCs	3D collagen Type I substrate	Enhanced cardiogenic gene expression compared to TCP coated with collagen Type I or laminin, which induced cardiogenic, osteogenic and adipogenic gene expression	Jung et al., 2016 [103]
hMSCs	Collagen Type I 3D patch	Increased expression of α-MHC and cTnT	Rashedi et al., 2017 [104]
rCPCs	Porcine-derived cardiac decellularized ECM vs. collagen matrix	Enhanced proliferation, adhesion and apoptosis reduction. Increased expression of Nkx2.5, Gata-4, cTnT and α-MHC	French et al., 2012 [105]
hCPCs	Biomatrix obtained from adult human cardiac fibroblasts isolated from healthy and pathological heart tissues.	Biomatrix stimulated migration and protected cells from apoptotic processes.	Castaldo et al., 2013 [106]
hCPCs	Polyurethane-based scaffold	Scaffold supported in vitro CPC adhesion and viability; it did not support cell proliferation.	Chiono et al., 2013 [88]
hCPCs	3D collagen-based vs. porcine cardiac ECM-based hydrogel.	Cardiac ECM hydrogel enhanced Gata-4, Mlc2v, Vegfr2 expression in foetal CPCs and Nkx2.5, Mef2c, CD31, Vegfr2 expression in adult CPCs.	Gaetani et al., 2016 [107]
hCPC	3D PU-based scaffold with laminin-1 surface functionalization	Increased CPC proliferation, decreased CPC apoptosis. Expression of cardiac markers (Mef2c and α sarcomeric actinin), smooth muscle cell markers (Gata-6 and SMA) and endothelial cell markers (ETS1 and FVIII)	Boffito et al., 2018 [91]
Mouse embryonic fibroblasts (MEFs)	Indirect reprogramming through different ECM protein based-hydrogels (Matrigel, collagen Type I and fibrin)	Fibrin hydrogel supported both dedifferentiation and differentiation phase; collagen Type I /fibrin gel increased the percentage of contractile colonies.	Kong et al., 2013 [108]
MEFs	Direct reprogramming using PEG hydrogels functionalized with laminin and RGD at different concentration vs. TCP	High concentration of laminin and RGD supported MEF reprogramming	Smith et al., 2013 [109]
MEFs, Tail tip fibroblasts (TTFs)	miRNA mediated-direct reprogramming using fibrin based-3D hydrogels vs. 2D culture	Fibrin based-3D hydrogels supported MEF reprogramming enhancing α-MHC, cTnI, α-sarcomeric actinin and Kcnj2 expression; increased Mef2c, Tbx5 and Hand2 expression in TTFs.	Li et al., 2016 [110]

**Table 2:** Main biomaterial substrates favouring differentiation of stem cells or direct/indirect reprogramming of fibroblasts into CMs.

Cells	Biomaterials for CM Generation
**Stem cell differentiation**	
Pluripotent stem cells:	
ESCs	Decellularized cardiac ECM [93], fibrin /Matrigel hydrogel [92], collagen Type I [94]—fibronectin/laminin 70/30 hydrogel [95]
iPSCs	Decellularized cardiac ECM [97], gelatin [96]—and collagen Type I [98]-coated scaffolds/hydrogels
Multipotent stem cells:	
MSCs	Cardiogel [101], collagen Type V substrates [99]
CPCs	Decellularized cardiac ECM [105], biomatrix [106], laminin-1 coated scaffolds [91]
**Indirect Reprogramming**	
Fibroblasts	Collagen Type I/Fibrin and Fibrin hydrogels [108]
**Direct Reprogramming**	
Fibroblasts	Fibrin/Matrigel hydrogel [110], PEG hydrogel containing RGD and laminin [109]

## References

[1] Addis R.C., Epstein J.A. (2013). Induced regeneration—The progress and promise of direct reprogramming for heart repair. Nat. Med..

[2] Chen Q.-Z., Harding S.E., Ali N.N., Lyon A.R., Boccaccini A.R. (2008). Biomaterials in cardiac tissue engineering: Ten years of research survey. Mater. Sci. Eng. R Rep..

[3] Curley D., Lavin Plaza B., Shah A.M., Botnar R.M. (2018). Molecular imaging of cardiac remodelling after myocardial infarction. Basic Res. Cardiol..

[4] Fraccarollo D., Galuppo P., Bauersachs J. (2012). Novel therapeutic approaches to post-infarction remodelling. Cardiovasc. Res..

[5] Pavesi A., Adriani G., Rasponi M., Zervantonakis I.K., Fiore G.B., Kamm R.D. (2015). Controlled electromechanical cell stimulation on-a-chip. Sci. Rep..

[6] Segers V.F.M., Lee R.T. (2008). Stem-cell therapy for cardiac disease. Nature.

[7] Ponikowski P., Anker S.D., AlHabib K.F., Cowie M.R., Force T.L., Hu S., Jaarsma T., Krum H., Rastogi V., Rohde L.E. (2014). Heart failure: Preventing disease and death worldwide. ESC Heart Fail..

[8] Silvestri A., Boffito M., Sartori S., Ciardelli G. (2013). Biomimetic Materials and Scaffolds for Myocardial Tissue Regeneration. Macromol. Biosci..

[9] Radhakrishnan J., Krishnan U.M., Sethuraman S. (2014). Hydrogel based injectable scaffolds for cardiac tissue regeneration. Biotechnol. Adv..

[10] Sanganalmath S.K., Bolli R. (2013). Cell Therapy for Heart Failure. Circ. Res..

[11] Hodgkinson C.P., Bareja A., Gomez J.A., Dzau V.J. (2016). Emerging Concepts in Paracrine Mechanisms in Regenerative Cardiovascular Medicine and Biology. Circ. Res..

[12] Batty J.A., Lima J.A.C., Kunadian V. (2016). Direct cellular reprogramming for cardiac repair and regeneration. Eur. J. Heart Fail..

[13] Doppler S., Deutsch M.-A., Lange R., Krane M. (2015). Direct Reprogramming—The Future of Cardiac Regeneration?. Int. J. Mol. Sci..

[14] Nam Y.-J., Song K., Olson E.N. (2013). Heart repair by cardiac reprogramming. Nat. Med..

[15] Hodgkinson C.P., Kang M.H., Dal-Pra S., Mirotsou M., Dzau V.J. (2015). MicroRNAs and Cardiac Regeneration. Circ. Res..

[16] Ebrahimi B. (2017). In vivo reprogramming for heart regeneration: A glance at efficiency, environmental impacts, challenges and future directions. J. Mol. Cell. Cardiol..

[17] Duelen R., Sampaolesi M. (2017). Stem Cell Technology in Cardiac Regeneration: A Pluripotent Stem Cell Promise. EBioMedicine.

[18] Thomson J.A., Itskovitz-Eldor J., Shapiro S.S., Waknitz M.A., Swiergiel J.J., Marshall V.S., Jones J.M. (1998). Embryonic stem cell lines derived from human blastocysts. Science.

[19] Chong J.J.H., Yang X., Don C.W., Minami E., Liu Y.-W., Weyers J.J., Mahoney W.M., Van Biber B., Cook S.M., Palpant N.J. (2014). Human embryonic-stem-cell-derived cardiomyocytes regenerate non-human primate hearts. Nature.

[20] Hartman M.E., Dai D.-F., Laflamme M.A. (2016). Human pluripotent stem cells: Prospects and challenges as a source of cardiomyocytes for in vitro modeling and cell-based cardiac repair. Adv. Drug Deliv. Rev..

[21] Takahashi K., Yamanaka S. (2006). Induction of Pluripotent Stem Cells from Mouse Embryonic and Adult Fibroblast Cultures by Defined Factors. Cell.

[22] Singh V.K., Kalsan M., Kumar N., Saini A., Chandra R. (2015). Induced pluripotent stem cells: Applications in regenerative medicine, disease modeling, and drug discovery. Front. Cell Dev. Biol..

[23] Rojas S.V., Kensah G., Rotaermel A., Baraki H., Kutschka I., Zweigerdt R., Martin U., Haverich A., Gruh I., Martens A. (2017). Transplantation of purified iPSC-derived cardiomyocytes in myocardial infarction. PLoS ONE.

[24] Casini S., Verkerk A.O., Remme C.A. (2017). Human iPSC-Derived Cardiomyocytes for Investigation of Disease Mechanisms and Therapeutic Strategies in Inherited Arrhythmia Syndromes: Strengths and Limitations. Cardiovasc. Drugs Ther..

[25] Singh A., Singh A., Sen D. (2016). Mesenchymal stem cells in cardiac regeneration: A detailed progress report of the last 6 years (2010–2015). Stem Cell Res. Ther..

[26] Chamberlain G., Fox J., Ashton B., Middleton J. (2007). Concise Review: Mesenchymal Stem Cells: Their Phenotype, Differentiation Capacity, Immunological Features, and Potential for Homing. Stem Cells.

[27] Miao C., Lei M., Hu W., Han S., Wang Q. (2017). A brief review: The therapeutic potential of bone marrow mesenchymal stem cells in myocardial infarction. Stem Cell Res. Ther..

[28] Rosland G.V., Svendsen A., Torsvik A., Sobala E., McCormack E., Immervoll H., Mysliwietz J., Tonn J.-C., Goldbrunner R., Lonning P.E. (2009). Long-term Cultures of Bone Marrow-Derived Human Mesenchymal Stem Cells Frequently Undergo Spontaneous Malignant Transformation. Cancer Res..

[29] Mauretti A., Spaans S., Bax N.A.M., Sahlgren C., Bouten C.V.C. (2017). Cardiac Progenitor Cells and the Interplay with Their Microenvironment. Stem Cells Int..

[30] Le T., Chong J. (2016). Cardiac progenitor cells for heart repair. Cell Death Discov..

[31] Zaruba M.M., Soonpaa M., Reuter S., Field L.J. (2010). Cardiomyogenic Potential of C-Kit+-Expressing Cells Derived from Neonatal and Adult Mouse Hearts. Circulation.

[32] Soonpaa M.H., Rubart M., Field L.J. (2013). Challenges measuring cardiomyocyte renewal. Biochim. Biophys. Acta.

[33] Leri A., Kajstura J., Anversa P. (2011). Role of Cardiac Stem Cells in Cardiac Pathophysiology: A Paradigm Shift in Human Myocardial Biology. Circ. Res..

[34] Anversa P., Kajstura J., Rota M., Leri A. (2013). Regenerating new heart with stem cells. J. Clin. Investig..

[35] Loughran J.H., Elmore J.B., Waqar M., Chugh A.R., Bolli R. (2012). Cardiac Stem Cells in Patients with Ischemic Cardiomyopathy: Discovery, Translation, and Clinical Investigation. Curr. Atheroscler. Rep..

[36] Dey D., Han L., Bauer M., Sanada F., Oikonomopoulos A., Hosoda T., Unno K., De Almeida P., Leri A., Wu J.C. (2013). Dissecting the Molecular Relationship Among Various Cardiogenic Progenitor Cells. Circ. Res..

[37] Hastings C.L., Roche E.T., Ruiz-Hernandez E., Schenke-Layland K., Walsh C.J., Duffy G.P. (2015). Drug and cell delivery for cardiac regeneration. Adv. Drug Deliv. Rev..

[38] van Berlo J.H., Kanisicak O., Maillet M., Vagnozzi R.J., Karch J., Lin S.-C.J., Middleton R.C., Marbán E., Molkentin J.D. (2014). c-kit+ cells minimally contribute cardiomyocytes to the heart. Nature.

[39] Qian L., Srivastava D. (2013). Direct Cardiac Reprogramming: From Developmental Biology to Cardiac Regeneration. Circ. Res..

[40] Chen Y., Yang Z., Zhao Z.-A., Shen Z. (2017). Direct reprogramming of fibroblasts into cardiomyocytes. Stem Cell Res. Ther..

[41] Huang C., Tu W., Fu Y., Wang J., Xie X. (2018). Chemical-induced cardiac reprogramming in vivo. Cell Res..

[42] Ieda M., Fu J.-D., Delgado-Olguin P., Vedantham V., Hayashi Y., Bruneau B.G., Srivastava D. (2010). Direct Reprogramming of Fibroblasts into Functional Cardiomyocytes by Defined Factors. Cell.

[43] Zhao Y., Londono P., Cao Y., Sharpe E.J., Proenza C., O’Rourke R., Jones K.L., Jeong M.Y., Walker L.A., Buttrick P.M. (2015). High-efficiency reprogramming of fibroblasts into cardiomyocytes requires suppression of pro-fibrotic signalling. Nat. Commun..

[44] Gnecchi M., Pisano F., Bariani R. (2015). MicroRNA and Cardiac Regeneration. Adv. Exp. Med. Biol..

[45] Jayawardena T.M., Egemnazarov B., Finch E.A., Zhang L., Payne J.A., Pandya K., Zhang Z., Rosenberg P., Mirotsou M., Dzau V.J. (2012). MicroRNA-Mediated In Vitro and In Vivo Direct Reprogramming of Cardiac Fibroblasts to Cardiomyocytes. Circ. Res..

[46] Lalit P.A., Salick M.R., Nelson D.O., Squirrell J.M., Shafer C.M., Patel N.G., Saeed I., Schmuck E.G., Markandeya Y.S., Wong R. (2016). Lineage Reprogramming of Fibroblasts into Proliferative Induced Cardiac Progenitor Cells by Defined Factors. Cell Stem Cell.

[47] Zhang R., Han P., Yang H., Ouyang K., Lee D., Lin Y.-F., Ocorr K., Kang G., Chen J., Stainier D.Y.R. (2013). In vivo cardiac reprogramming contributes to zebrafish heart regeneration. Nature.

[48] Giacca M., Zacchigna S. (2015). Harnessing the microRNA pathway for cardiac regeneration. J. Mol. Cell. Cardiol..

[49] Zacchigna S., Giacca M. (2014). Extra- and intracellular factors regulating cardiomyocyte proliferation in postnatal life. Cardiovasc. Res..

[50] Zacchigna S., Martinelli V., Moimas S., Colliva A., Anzini M., Nordio A., Costa A., Rehman M., Vodret S., Pierro C. (2018). Paracrine effect of regulatory T cells promotes cardiomyocyte proliferation during pregnancy and after myocardial infarction. Nat. Commun..

[51] Scuderi G.J., Butcher J. (2017). Naturally Engineered Maturation of Cardiomyocytes. Front. Cell Dev. Biol..

[52] McCulley D.J., Black B.L. (2012). Transcription factor pathways and congenital heart disease. Curr. Top. Dev. Biol..

[53] He A., Gu F., Hu Y., Ma Q., Ye L.Y., Akiyama J.A., Visel A., Pennacchio L.A., Pu W.T. (2014). Dynamic GATA4 enhancers shape the chromatin landscape central to heart development and disease. Nat. Commun..

[54] Bai F., Ho Lim C., Jia J., Santostefano K., Simmons C., Kasahara H., Wu W., Terada N., Jin S. (2015). Directed Differentiation of Embryonic Stem Cells into Cardiomyocytes by Bacterial Injection of Defined Transcription Factors. Sci. Rep..

[55] Kolanowski T.J., Antos C.L., Guan K. (2017). Making human cardiomyocytes up to date: Derivation, maturation state and perspectives. Int. J. Cardiol..

[56] Srivastava D. (2006). Making or Breaking the Heart: From Lineage Determination to Morphogenesis. Cell.

[57] Robertson C., Tran D.D., George S.C. (2013). Concise review: Maturation phases of human pluripotent stem cell-derived cardiomyocytes. Stem Cells.

[58] Yang X., Pabon L., Murry C.E. (2014). Engineering Adolescence: Maturation of Human Pluripotent Stem Cell-Derived Cardiomyocytes. Circ. Res..

[59] Bergmann O., Zdunek S., Alkass K., Druid H., Bernard S., Frisén J. (2011). Identification of cardiomyocyte nuclei and assessment of ploidy for the analysis of cell turnover. Exp. Cell Res..

[60] McCain M.L., Parker K.K. (2011). Mechanotransduction: The role of mechanical stress, myocyte shape, and cytoskeletal architecture on cardiac function. Pflüg. Arch. Eur. J. Physiol..

[61] Manfra O., Frisk M., Louch W.E. (2017). Regulation of Cardiomyocyte T-Tubular Structure: Opportunities for Therapy. Curr. Heart Fail. Rep..

[62] Zhu H., Scharnhorst K.S., Stieg A.Z., Gimzewski J.K., Minami I., Nakatsuji N., Nakano H., Nakano A. (2017). Two dimensional electrophysiological characterization of human pluripotent stem cell-derived cardiomyocyte system. Sci. Rep..

[63] Angst B.D., Khan L.U., Severs N.J., Whitely K., Rothery S., Thompson R.P., Magee A.I., Gourdie R.G. (1997). Dissociated spatial patterning of gap junctions and cell adhesion junctions during postnatal differentiation of ventricular myocardium. Circ. Res..

[64] Wang H., Xi Y., Zheng Y., Wang X., Cooney A.J. (2016). Generation of electrophysiologically functional cardiomyocytes from mouse induced pluripotent stem cells. Stem Cell Res..

[65] Piquereau J., Caffin F., Novotova M., Lemaire C., Veksler V., Garnier A., Ventura-Clapier R., Joubert F. (2013). Mitochondrial dynamics in the adult cardiomyocytes: Which roles for a highly specialized cell?. Front. Physiol..

[66] Zhou Y., Wang L., Liu Z., Alimohamadi S., Yin C., Liu J., Qian L. (2017). Comparative Gene Expression Analyses Reveal Distinct Molecular Signatures between Differentially Reprogrammed Cardiomyocytes. Cell Rep..

[67] Kashyap V., Rezende N.C., Scotland K.B., Shaffer S.M., Persson J.L., Gudas L.J., Mongan N.P. (2009). Regulation of Stem Cell Pluripotency and Differentiation Involves a Mutual Regulatory Circuit of the Nanog, OCT4, and SOX2 Pluripotency Transcription Factors with Polycomb Repressive Complexes and Stem Cell microRNAs. Stem Cells Dev..

[68] Gattazzo F., Urciuolo A., Bonaldo P. (2014). Extracellular matrix: A dynamic microenvironment for stem cell niche. Biochim. Biophys. Acta Gen. Subj..

[69] Chen S.S., Fitzgerald W., Zimmerberg J., Kleinman H.K., Margolis L. (2007). Cell-Cell and Cell-Extracellular Matrix Interactions Regulate Embryonic Stem Cell Differentiation. Stem Cells.

[70] Engler A.J., Sen S., Sweeney H.L., Discher D.E. (2006). Matrix Elasticity Directs Stem Cell Lineage Specification. Cell.

[71] Wang H.-B., Dembo M., Wang Y.-L. (2000). Substrate flexibility regulates growth and apoptosis of normal but not transformed cells. Am. J. Physiol. Physiol..

[72] Miskon A., Mahara A., Uyama H., Yamaoka T. (2010). A suspension induction for myocardial differentiation of rat mesenchymal stem cells on various extracellular matrix proteins. Tissue Eng. Part C Methods.

[73] Lockhart M., Wirrig E., Phelps A., Wessels A. (2011). Extracellular matrix and heart development. Birth Defects Res. Part A Clin. Mol. Teratol..

[74] Xi J., Khalil M., Shishechian N., Hannes T., Pfannkuche K., Liang H., Fatima A., Haustein M., Suhr F., Bloch W. (2010). Comparison of contractile behavior of native murine ventricular tissue and cardiomyocytes derived from embryonic or induced pluripotent stem cells. FASEB J..

[75] Marinkovic M., Block T.J., Rakian R., Li Q., Wang E., Reilly M.A., Dean D.D., Chen X.-D. (2016). One size does not fit all: Developing a cell-specific niche for in vitro study of cell behavior. Matrix Biol..

[76] Martino S., D’Angelo F., Armentano I., Kenny J.M., Orlacchio A. (2012). Stem cell-biomaterial interactions for regenerative medicine. Biotechnol. Adv..

[77] Ikonen L. (2013). Analysis of Different Natural and Synthetic Biomaterials to Support Cardiomyocyte Growth. J. Clin. Exp. Cardiol..

[78] Morez C., Noseda M., Abreu M., Belian E., Schneider M.D., Stevens M.M. (2015). Biomaterials Enhanced ef fi ciency of genetic programming toward cardiomyocyte creation through topographical cues. Biomaterials.

[79] Engler A.J., Carag-Krieger C., Johnson C.P., Raab M., Tang H.-Y., Speicher D.W., Sanger J.W., Sanger J.M., Discher D.E. (2008). Embryonic cardiomyocytes beat best on a matrix with heart-like elasticity: Scar-like rigidity inhibits beating. J. Cell Sci..

[80] Yang G., Xiao Z., Ren X., Long H., Ma K., Qian H., Guo Y. (2017). Obtaining spontaneously beating cardiomyocyte-like cells from adipose-derived stromal vascular fractions cultured on enzyme-crosslinked gelatin hydrogels. Sci. Rep..

[81] Cutts J., Nikkhah M., Brafman D.A. (2015). Biomaterial Approaches for Stem Cell-Based Myocardial Tissue Engineering. Biomark. Insights.

[82] Lee S.-W., Lee H.J., Hwang H.S., Ko K., Han D.W., Ko K. (2015). Optimization of Matrigel-based culture for expansion of neural stem cells. Anim. Cells Syst..

[83] Hinderer S., Layland S.L., Schenke-Layland K. (2016). ECM and ECM-like materials—Biomaterials for applications in regenerative medicine and cancer therapy. Adv. Drug Deliv. Rev..

[84] Moroni F., Mirabella T. (2014). Decellularized matrices for cardiovascular tissue engineering. Am. J. Stem Cells.

[85] Chachques J.C., Pradas M.M., Bayes-Genis A., Semino C. (2013). Creating the bioartificial myocardium for cardiac repair: Challenges and clinical targets. Expert Rev. Cardiovasc. Ther..

[86] Lakshmanan R., Krishnan U.M., Sethuraman S. (2013). Polymeric Scaffold Aided Stem Cell Therapeutics for Cardiac Muscle Repair and Regeneration. Macromol. Biosci..

[87] Sartori S., Chiono V., Tonda-Turo C., Mattu C., Gianluca C. (2014). Biomimetic polyurethanes in nano and regenerative medicine. J. Mater. Chem. B.

[88] Chiono V., Mozetic P., Boffito M., Sartori S., Gioffredi E., Silvestri A., Rainer A., Giannitelli S.M., Trombetta M., Nurzynska D. (2013). Polyurethane-based scaffolds for myocardial tissue engineering. Interface Focus.

[89] Carmagnola I., Ranzato E., Chiono V. (2018). Scaffold functionalization to support a tissue biocompatibility. Functional 3D Tissue Engineering Scaffolds.

[90] Chiono V., Nardo T., Ciardelli G. (2014). Bioartificial materials for regenerative medicine applications. Regenerative Medicine Applications in Organ Transplantation.

[91] Boffito M., Di Meglio F., Mozetic P., Giannitelli S.M., Carmagnola I., Castaldo C., Nurzynska D., Sacco A.M., Miraglia R., Montagnani S. (2018). Surface functionalization of polyurethane scaffolds mimicking cardiac microenvironment to support cardiac primitive cells. PLoS ONE.

[92] Zhang D., Shadrin I.Y., Lam J., Xian H.-Q., Snodgrass H.R., Bursac N. (2013). Tissue-engineered cardiac patch for advanced functional maturation of human ESC-derived cardiomyocytes. Biomaterials.

[93] Higuchi S., Lin Q., Wang J., Lim T.K., Joshi S.B., Anand G.S., Chung M.C.M., Sheetz M.P., Fujita H. (2013). Heart extracellular matrix supports cardiomyocyte differentiation of mouse embryonic stem cells. J. Biosci. Bioeng..

[94] Prabhakaran M.P., Mobarakeh L.G., Kai D., Karbalaie K., Nasr-Esfahani M.H., Ramakrishna S. (2014). Differentiation of embryonic stem cells to cardiomyocytes on electrospun nanofibrous substrates. J. Biomed. Mater. Res. Part B Appl. Biomater..

[95] Sa S., Wong L., McCloskey K.E. (2014). Combinatorial Fibronectin and Laminin Signaling Promote Highly Efficient Cardiac Differentiation of Human Embryonic Stem Cells. BioRes. Open Access.

[96] Chen Y., Zeng D., Ding L., Li X.L., Liu X.T., Li W.J., Wei T., Yan S., Xie J.H., Wei L. (2015). Three-dimensional poly-(ε-caprolactone) nanofibrous scaffolds directly promote the cardiomyocyte differentiation of murine-induced pluripotent stem cells through Wnt/β-catenin signaling. BMC Cell Biol..

[97] Fong A.H., Romero-López M., Heylman C.M., Keating M., Tran D., Sobrino A., Tran A.Q., Pham H.H., Fimbres C., Gershon P.D. (2016). Three-Dimensional Adult Cardiac Extracellular Matrix Promotes Maturation of Human Induced Pluripotent Stem Cell-Derived Cardiomyocytes. Tissue Eng. Part A.

[98] Hirata M., Yamaoka T. (2018). Effect of stem cell niche elasticity/ECM protein on the self-beating cardiomyocyte differentiation of induced pluripotent stem (iPS) cells at different stages. Acta Biomater..

[99] Tan G., Shim W., Gu Y., Qian L., Ying Chung Y., Yun Lim S., Yong P., Sim E., Wong P. (2010). Differential effect of myocardial matrix and integrins on cardiac differentiation of human mesenchymal stem cells. Differentiation.

[100] Guan J., Wang F., Li Z., Chen J., Guo X., Liao J., Moldovan N.I. (2011). The stimulation of the cardiac differentiation of mesenchymal stem cells in tissue constructs that mimic myocardium structure and biomechanics. Biomaterials.

[101] Santhakumar R., Vidyasekar P., Verma R.S. (2014). Cardiogel: A Nano-Matrix Scaffold with Potential Application in Cardiac Regeneration Using Mesenchymal Stem Cells. PLoS ONE.

[102] Park J., Park S., Ryu S., Bhang S.H., Kim J., Yoon J.-K., Park Y.H., Cho S.-P., Lee S., Hong B.H. (2014). Graphene-Regulated Cardiomyogenic Differentiation Process of Mesenchymal Stem Cells by Enhancing the Expression of Extracellular Matrix Proteins and Cell Signaling Molecules. Adv. Healthc. Mater..

[103] Jung J.P., Bache-Wiig M.K., Provenzano P.P., Ogle B.M. (2016). Heterogeneous Differentiation of Human Mesenchymal Stem Cells in 3D Extracellular Matrix Composites. BioRes. Open Access.

[104] Rashedi I., Talele N., Wang X.-H., Hinz B., Radisic M., Keating A. (2017). Collagen scaffold enhances the regenerative properties of mesenchymal stromal cells. PLoS ONE.

[105] French K.M., Boopathy A.V., DeQuach J.A., Chingozha L., Lu H., Christman K.L., Davis M.E. (2012). A naturally derived cardiac extracellular matrix enhances cardiac progenitor cell behavior in vitro. Acta Biomater..

[106] Castaldo C., Di Meglio F., Miraglia R., Sacco A.M., Romano V., Bancone C., Della Corte A., Montagnani S., Nurzynska D. (2013). Cardiac Fibroblast-Derived Extracellular Matrix (Biomatrix) as a Model for the Studies of Cardiac Primitive Cell Biological Properties in Normal and Pathological Adult Human Heart. BioMed Res. Int..

[107] Gaetani R., Yin C., Srikumar N., Braden R., Doevendans P.A., Sluijter J.P.G., Christman K.L. (2016). Cardiac-Derived Extracellular Matrix Enhances Cardiogenic Properties of Human Cardiac Progenitor Cells. Cell Transplant..

[108] Kong Y.P., Carrion B., Singh R.K., Putnam A.J. (2013). Matrix identity and tractional forces influence indirect cardiac reprogramming. Sci. Rep..

[109] Smith A.W., Hoyne J.D., Nguyen P.K., McCreedy D.A., Aly H., Efimov I.R., Rentschler S., Elbert D.L. (2013). Direct reprogramming of mouse fibroblasts to cardiomyocyte-like cells using Yamanaka factors on engineered poly(ethylene glycol) (PEG) hydrogels. Biomaterials.

[110] Li Y., Dal-Pra S., Mirotsou M., Jayawardena T.M., Hodgkinson C.P., Bursac N., Dzau V.J. (2016). Tissue-engineered 3-dimensional (3D) microenvironment enhances the direct reprogramming of fibroblasts into cardiomyocytes by microRNAs. Sci. Rep..

[111] Vanwinkle W.B., Snuggs M.B., Buja L.M. (1996). Cardiogel: A biosynthetic extracellular matrix for cardiomyocyte culture. In Vitro Cell. Dev. Biol. Anim..

[112] Yuan T., Li K., Guo L., Fan H., Zhang X. (2011). Modulation of immunological properties of allogeneic mesenchymal stem cells by collagen scaffolds in cartilage tissue engineering. J. Biomed. Mater. Res. A.

[113] Qian L., Huang Y., Spencer C.I., Foley A., Vedantham V., Liu L., Conway S.J., Fu J.D., Srivastava D. (2012). In vivo reprogramming of murine cardiac fibroblasts into induced cardiomyocytes. Nature.

[114] Schuldiner M., Yanuka O., Itskovitz-Eldor J., Melton D.A., Benvenisty N. (2000). Effects of eight growth factors on the differentiation of cells derived from human embryonic stem cells. Proc. Natl. Acad. Sci. USA.

[115] Monaghan M.G., Holeiter M., Layland S.L., Schenke-Layland K. (2016). Cardiomyocyte generation from somatic sources—Current status and future directions. Curr. Opin. Biotechnol..

[116] Miner J.H., Yurchenco P.D. (2004). Laminin Functions In Tissue Morphogenesis. Annu. Rev. Cell Dev. Biol..

[117] Castaldo C., Di Meglio F., Nurzynska D., Romano G., Maiello C., Bancone C., Müller P., Böhm M., Cotrufo M., Montagnani S. (2008). CD117-Positive Cells in Adult Human Heart Are Localized in the Subepicardium, and Their Activation Is Associated with Laminin-1 and α_6_ Integrin Expression. Stem Cells.

[118] Stoppel W.L., Kaplan D.L., Black L.D. (2016). Electrical and mechanical stimulation of cardiac cells and tissue constructs. Adv. Drug Deliv. Rev..

